# Health promotion for blood donors: A scoping review

**DOI:** 10.1016/j.puhip.2025.100604

**Published:** 2025-03-22

**Authors:** Linda J.H. Marx, Suzanna M. Van Walraven, Ruben van Zelm, Barbara Sassen

**Affiliations:** a Sanquin Blood Supply Foundation, Plesmanlaan 125, 1066 CX, Amsterdam, the Netherlands; b University of Applied Sciences, Heidelberglaan 7, 3584 CS, Utrecht, the Netherlands

**Keywords:** Donor health, Blood banks, Health promotion, Public health, Barriers to implementation, Quadruple aim approach

## Abstract

**Background:**

Health promotion aims to prevent chronic diseases and alleviate their impact on individuals' well-being. It involves empowering both individuals and communities to actively uphold optimal health. Although blood banks conventionally centre on blood donation functions, an untapped potential lies in leveraging these institutions for health enhancement through prioritized health promotion initiatives. The objective of this study is to explore how blood banks can contribute to the well-being of blood donors by integrating preventive measures as part of health promotion initiatives within their operations.

**Study design:**

Literature review.

**Methods:**

Literature Search in CINAHL, Embase, PubMed and Web of Science was conducted. Arksey and O'Malley (2015) framework was used with incorporated recommendations stated by Levac, Colquhoun, and O'Brien (2010) following an iterative process of six stages, starting with identifying the research question, identifying relevant studies, study selection, charting the data, collating, summarizing, and reporting the results, and consult experts.

**Results:**

The 20 incorporated studies showed a range of health screenings and health promoting activities, that expand the traditional scope of blood bank addressing blood-related conditions, encompassing assessments for conditions related to blood as well as health screenings. These screenings included examinations for cancer and diabetes, with the predominant focus being on cardiovascular risk assessment.

**Conclusion:**

This review highlights the implementation of health initiatives by blood banks, focusing on improving well-being and preventing diseases. These initiatives have the potential to act as gateways for community-based interventions and possibilities for enhancing both the blood supply and individual health. The effectiveness of these interventions is contingent upon the context of each blood bank and its target donor group. Therefore, tailoring interventions to align with specific contexts and facilitating factors is crucial for optimizing health promotion efforts tailored to the diverse needs of different donor groups.

## 1 Introduction

Blood transfusion saves lives and improves health [1]. According to the World Health Organization (WHO) a safe and sufficient blood supply must be ensured by every country's health care policy [1]. Blood banks depend on the commitment of blood donors to meet the demand and supply of blood products [1]. In line with the paradigm shift in health promotion initiated by the Ottawa Charter in 1986 [2], blood banks contributing to public health align with the principles of community health approaches and empowering individuals.

Health promotion, as defined by the WHO, enables people to improve their health [3], encompassing social and environmental aspects [4]. The 2016 global conference on health promotion emphasized sustainable development goals [4]. When implementing health promotion and disease prevention, blood banks can expand their role from being a place solely for blood donation to becoming important for assessing, monitoring, and improving individual health, thereby contributing to public health [5].

Moreover, blood banks have contact with large number of people annually, providing them screening opportunities [6]. Despite the “healthy donor effect", where donors in general report better self-rated health compared to the general population, risk factors are also present among donors. This includes for example hyperglycaemia and overweight [[7], [8], [9], [10]]. By addressing these risk-factors, blood banks could not only promote the health of blood donors. By incorporating health promotion and prevention, blood banks can help improve public health outcomes and lower healthcare costs [11. Expanding the role of blood banks in prevention is imperative, especially as chronic diseases continue to rise enabling them to significantly impact (individuals') well-being and reduce healthcare costs [11,12].

Health promotion for blood donors benefits the individual donor, and may improve blood product quality, as it is impacted by health status, smoking, and alcohol consumption [[13], [14], [15], [16]]. Blood banks could improve donor experience and health, improving population health, and reducing costs, aligning with "triple aim" proposed by Berwick et al. (2008) [17,18].

While many articles describe health interventions piloted in blood banks, there is a literature gap for when and how these interventions can be successfully brought into practice. The objective of this study is to investigate the potential of blood banks to enhance the well-being of blood donors through the implementation of preventive measures and health promotion initiatives in blood banks. The research question for this scoping review is stated as; which health initiatives are implemented by blood banks to promote health or prevent diseases of donors?

## 2 Methods

In this scoping review we followed the framework stating an iterative six-stage process, initially developed by Arksey and O'Malley (2005) [19] and incorporates recommendations stated by Levac, Colquhoun, and O'Brien (2010) [20]. This framework facilitated the identification of concepts, research gaps, and diverse types and sources of evidence.

### 2.1 Stage 1: Identifying the research question

The initial step involved employing the PICO-method to construct a search rule and perform an initial search [21]. The search focused on blood banks(P) using interventions(I) that (could potentially) promote the health of blood donors(O). After the initial search, we included keywords in the search rule, found in these studies. The search strings consist of MeSH terms, obtained from the NCBI's Medical Subject Heading database (used in PubMed), and relevant terms in titles and abstract.

### 2.2 Stage 2: Identifying relevant studies

We conducted our literature search using validated search rules. The search terms and operators were adapted to the specific database's requirements. Adaptations to search terms and operators were made, according to database requirements and functions (see Appendix 1). Additionally, the reference lists of retrieved articles were meticulously examined, and the citation indexes of Google Scholar were utilized to identify additional relevant studies, following the snowball sampling method described by Gough, Oliver, and Thomas (2012) [22]. We conducted searches from February 2022 to May 2023, using publications limited to those in English.

### 2.3 Stage 3: Study selection

The selection criteria were based on the PRISMA 2020 Statement published in 2021 [23]. Studies focused on health promotion or disease prevention for blood donors, were selected. Studies that focused on health promotion as part of the donor selection process, or solely aimed on enhancing blood donations by offering health checks, were not included. Articles concerning hemochromatosis and iron overload screening, as well as those focused on the willingness of donors to provide data for research benefits and studies related to the storage of health data intended for research purposes, biobanking and genetic screening by blood banks, were not selected. If articles were not full-text accessible (abstracts or congress flyers), we did not select them. We evaluated the quality of the articles and assessed their validity and reliability (see Appendix 2). Based on the quality assessment, using critical appraisal tools relevant for methodology and design of the study, we included articles with sufficient quality. Thus the quality of prospective controlled trials, retrospective, cohort, observational and cross-sectional studies was determined by using the Strengthening the Reporting of Observational Studies in Epidemiology (STROBE) [24]. The quality of articles such as a letter to the editor, status reports and opinion articles were assessed by utilizing expert opinion. See Appendix 2 for detailed information regarding the appraisal process.

### 2.4 Stage 4: Charting the data

Information collected from the articles stated first author, year of publication, country, study design employed, study population and intervention, main findings of the study, and implementation status of the health initiatives mentioned. To streamline the data collection process, the authors, titles and publication date of the identified articles were uploaded into an Excel spreadsheet. Article was flagged if it was excluded from the review, along with the reasons for exclusion. Also, citation of authors that were related to the research question were included in the spreadsheet.

Furthermore, additional details were recorded, such as citation, a digital link to the journal article, the source's design (e.g., retrospective cohort study, cross-sectional study), and descriptions or definitions of the donor data used to promote donors' health. Also noted were descriptions or definitions of health promotion and the target group (e.g., USA donors). For the ease of readers, and to create clarity and unambiguity, the articles were sorted based on date of first publication. If several articles were published in the same year, the articles were also sorted by alphabet.

### 2.5 Stage 5: Collating, summarizing and reporting the results

Levac et al. (2010) [20] recommend thematic construction to provide an overview of the breadth of the literature. We therefore presented a thematic analysis according to the themes that were reoccurring, namely health screenings, community-based health intervention, and healthcare context, tailoring and demographics.

### 2.6 Stage 6: Consultations

Following the guidelines proposed by Levac et al. (2010) [20], expert consultation was utilized to obtain more insight in health initiatives that are implemented by blood banks. We did this by approaching all mentioned corresponding authors and relations in our personal network.

## 3 Results

**Fig. 1:**
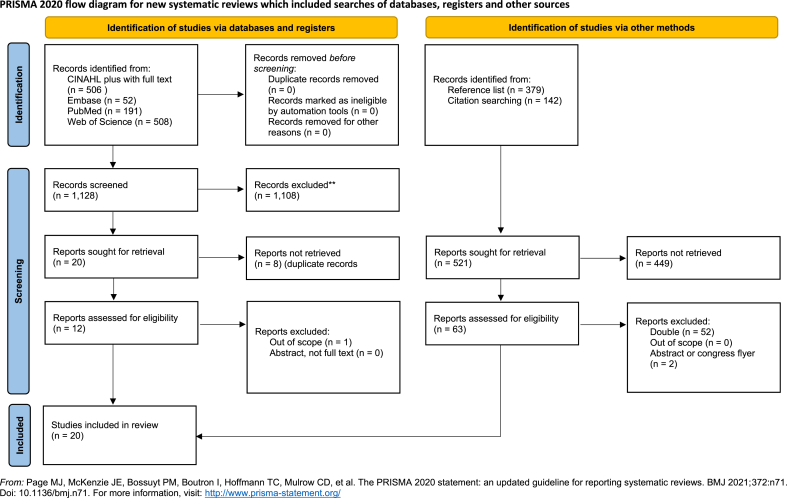
PRISMA flow diagram for search.

Following the Arksey and O'Malley framework, in stage 2 we conducted a literature search focused on blood banks using interventions with the aim to promote health of blood donors. We started formulating our research question and developing a search rule using the PICO-method. We searched in databanks and used reference lists to identify relevant studies. The selection of studies was based on PRISMA 2000 Statement. We selected studies that focused on health promotion or disease prevention for blood donors. Using this method, we identified 1,128 records from CINAHL, Embase, PubMed, and Web of Science. In this initial search we have included 11 records: [[25], [26], [27], [28], [29], [30], [31], [32], [33], [34], [35]]. After a search in the references and citations list of the first 11 included records, we were able to find an additional nine records: [5,6,[36], [37], [38], [39], [40], [41], [42]]. See Fig. 1 to see the search results in more details. We used appraisal tools to address the quality of the selected studies (stage 3). Based on the quality assessment, using the appraisal tools, we did not exclude any articles. The quality of the studies was as such we included them all. In total we selected 20 studies that focused on health initiatives with the aim of promoting donors' health and/or preventing disease, interventions piloted or implemented by blood banks. According to stage 4 of Marksey and O‘Malley framework, we charted the data. The characteristics of health promotion identified in the twenty publications are summarized in Table 1.

**Table 1:** Included after reading full text (organized by time of publishing).

	Author(s) and year	Disease identity or health status (including the number of donors screened, if applicable)	Country	Study design	Study population and intervention	Main findings	Is the intervention structurally implemented?
1.	(Hart et al., 1996) [25]	Collateral cancer (n = 556)	UK	Observational study	The study design involved promoting cancer screening, specifically colorectal cancer screening, to blood donors aged 51–65 years at the Leicester Blood Donor Centre [25].	- The study aimed to measure the ability of the blood transfusion service to promote cancer screening using the uptake of colorectal cancer screening offered to blood donors [25]. - Blood donors (n = 556) aged 51–65 years got information about collateral cancer screening. - The study was conducted over a period of 3 months. - Among the “subjects who arrived to donate blood and accepted screening, 63 % completed a faecal occult blood test” [25]. - There was a similar compliance in men and women [25]. - “The proportion of the total target population screened, including those donors invited to give blood but who did not attend, was 21 percent "[25]. - “The study successfully targeted donors who attended to give blood, but only a small proportion of the total target population was screened" [25]. - The presence of a doctor at the blood donor centre to explain the screening procedure contributed to the high acceptance rate among donors. - Compliance rates were similar between men and women who took the screening kits home, contrary to general practice studies where compliance is usually higher in women. - Older donors participated more than younger donors, suggesting that “direct contact and explanations to members of low-compliance groups increase their response" [25].	• Not mentioned • The study highlighted “the potential of blood donor centres to “display cancer screening and health promotion literature and encourage doctors supervising sessions to provide further information and advice to donors [25].
2.	(Davey, 2006) [5]	Health screenings for blood pressure hemoglobin, cholesterol, glucose, and other screenings like PSA and genetic testing.	USA	Status report or opinion article.	Not applicable: it's a status report: Davey (2006) mentions however following: • A total of 5432 donors were evaluated and 374 were found to have blood pressure readings in the hypertensive range. • Davey (2006) mentions the following optional screening tests; complete blood count, ferritin, total cholesterol, Prostate Specific Antigen, Glucose, and haemoglobin A1c, C-reactive protein. Other screening options, such as PSA and genetic testing, are seen as too controversial and costly for most blood centres to consider according to Davey (2006).	Recommended procedures and tests for blood bank intervention include: • Blood Pressure: Blood banks should have an education, counselling, and referral programme to address elevated blood pressure in donors. • Haemoglobin: Donors who fail the haemoglobin screening test should be informed about its significance. Blood banks may offer a complete blood count for those who do not pass the screening, especially if red cell indices suggest iron deficiency. Referral to a primary physician for further evaluation, including serum ferritin determination, may be appropriate. Other abnormal findings on the complete blood count should also prompt referrals. • Cholesterol: Offering an inexpensive optional cholesterol test can help donors address cardiovascular risk. Blood banks can provide an educational leaflet or brochure with the test results, guiding donors to consult their personal physician for further tests like a lipid profile, high-sensitivity C-reactive protein, and homocysteine [5]. • Glucose: Donors with random glucose levels exceeding 200 mg/dL should be advised to consult their personal physician for further evaluation. Fasting blood glucose levels can establish a diagnosis of diabetes if present, and haemoglobin A1c determinations can monitor blood glucose control. • Other screening options, such as PSA and genetic testing, are controversial and costly for most blood banks.	• Davey (2006) mentions that NYBC has adopted the hypertension education and counselling programme for all NYBC blood donors. • Many donors found the cholesterol test useful, and the NYBC now offers the test as an option at blood drives where it is requested by the drive organizers. • Davey (2006) states that providing screening and counselling for basic measures of good health can benefit both the donor and the community.
3.	(Longo, Lucci, Marconi & Cremonesi, 2007) [36]	Cardiovascular Disease Risk factors (n = 11.093)	Italy	Cross-sectional epidemiological study	• Longo et al. (2007) wanted to detect if donors had high cardiovascular risk scores. • Longo et al. (2007) screened total cholesterol, HDL cholesterol, blood sugar and the donor's BMI.	• Longo et al. (2007) identified a group of donors with a high cardiovascular risk score. • High percentage of smokers (about 23 %). • More than 50 % of the male donors were overweight (BMI> 25). • Blood pressure values showed about 30 % of the subjects were prehypertensive. • Overall, 75,4 % of the whole population were at low risk at CVD. • Donors will benefit from early identification.	• No, this study was part of the Project Heart Program of the Italian National Institute of Health.
4.	Dell'anna, Adorni, Bernuzzi Cantarelli, Cepparulo, Cocchi, Dell'anna, Formentini, Sassi, Scognamiglio, Vescovi, Franchini, 2010) [37]	Cardiovascular Disease Risk factors (n = 6.172)	Italy	Observational cross-sectional design	• “Between January 2007 and December 2008, 6.172 blood donors (aged 35–65 years)” participated in this study which calculated the donor's cardiovascular risk score [37].	• 5.8 % of the donors had a high cardiovascular risk score [37].[	• Dell’Anna et al. (2010) show that it is important to detect cardiovascular disease in a early stage. Dell’Anna et al. (2010) also state that implementing health screening for cardiovascular disease among donors is effective.
5.	(Eason, Goudar, Centilli, Sayers, 2011) [38]	Cholesterol (n = 187.714)	USA	Retrospective study	• The authors analysed the data from the routine total nonfasting blood cholesterol screening program that was implemented for volunteer blood and component donors at a blood bank in the US. • “Experience with screening 187.714 individual donors for total nonfasting cholesterol was reviewed and results were compared with those for a representative sample of United States adults as published in National Health and Nutrition Examination Surveys "[38].	• “Blood donation is seen not exclusively as an expression of altruism, but also as an opportunity for more general health promotion, particularly with regard to screening for cardiovascular disease risk and diabetes” [38]. “While knowledge of an individual's cholesterol is valuable to those at risk of cardiovascular disease, provided that the information prompts action when indicated, there are added benefits to considering a broader risk profile. This profile might include, for example, weight, blood sugar, abdominal girth, and blood pressure "[38]. • “The point has been made on several occasions that strategies are needed at many levels in the community, including schools and work sites, to promote healthy lifestyles. Strategies that combine donation with additional tests of general health are valuable in defining a role for blood programs to contribute to community health "[38]. • 9.3 % of males and 9.6 % of the females had cholesterol levels greater than 240 mg/d [38].	• Since 1995, the Border Blood Transfusion Service in South Africa asked additional questions about the health history prior to the donation, like diet, exercise, and alcohol intake, and additional testing. “A full blood count, total cholesterol, and prostate-specific antigen test (for men over 50) were performed and there was an optional urine screen for diabetes" [38]. • Cardiovascular risk assessment among donors has been explored as part of the Project Heart Program of the Italian National Institute of Health. In this study, donors are given cardiovascular risk scores calculated from assessments that include total cholesterol, HDL cholesterol, blood sugar, and body mass index" [38].
6.	(Mathai, Vasu Subhadramma, 2012) [26]	Hypertension (n = 18.452)	India	Retrospective observational study	• “Verification of records over a period of three years (2004-06), showed that out of a total of 18.452 donors, 951 were deferred and, among them 175 were due to hypertension. Thus about 0.95 % of healthy blood donors had undetected hypertension" [26].	• “About 1 % of healthy individuals were found to have undetected hypertension" [26]. • Identifying “risk factors among blood donors” can “expand the role of the blood bank in the community" [26]. • Observations from these studies justify extensive screening.	• Pilot study
7.	(Murphy et al., 2012) [27]	Obesitas (n = 1.042.817)	USA	Cross sectional study	The study population in the mentioned study by Murphy et al. (2012) consists of “blood donors at six blood banks across the United States" [27]. The blood banks are part of the “Retrovirus Epidemiology in Donors Study II (REDS-II), which is a multicentre consortium" [27]. The study includes data collected from January 2007 to December 2008, from all successful allogeneic blood donations made by unremunerated volunteer donors at these six REDS-II centres.	• A colanks have the potential to play a role in obesity and public health promotion [27].	• The study by Murphy et al. (2012) does not specifically mention whether the findings have been implemented or if it was a pilot study. • Blood banks can raise awareness by education, and promotion of weight management interventions among blood donors.
8.	(Shaz, Kessler, Hillyer, 2012) [28]	Disease like sickle cell disease & Health status, namely, diabetes screening, cholesterol testing, cardiovascular disease risk screening, genetic screening for hemochromatosis, iron replacement programs, influenza vaccination	USA	Status report/review	Status report “evaluating the role of blood centres in public health” [28].	• The studies identified and included in this review “do not support a significant improvement in donor health or significant increase in donation rates through these public health initiatives” [28]. A long-term study is required with adequate resources to obtain accurate data to truly evaluate the value of the blood centre's public health initiatives [28]. • Shaz et al., 2012 mention public health programs performed by blood banks like education (pamphlets including coping mechanisms for donation to avoid reactions, pamphlets and videos for African American communities about sickle cell disease), diagnostic testing for donors (cholesterol testing, diabetes screening (HbA1c), cardiovascular disease risk screening (body mass index, blood pressure, HbA1c, cholesterol level), genetic screening for hemochromatosis or treatments (hemochromatosis phlebotomy for use in allogeneic transfusion, iron replacement/supplementation programs) and a role in influenza vaccination [28].	• According to Shaz et al. (2012) no study has been carried out long enough to adequately evaluate the value of providing various health initiatives to blood donors [28].
9.	(Kessler, Ortiz, Grima, Vlahov, Nandi, Jones & Shaz, 2012) [29][29]	Cardiovascular Disease Risk factors (n = 2.406)	USA	Cross-sectional study.	• Over 11 months, 2.406 participants had CVD risk screening and counselling were performed at mobile 290 blood drives in diverse neighbourhoods. • Point of care testing, interviews and physical examination was performed.	• “Over 11 months, 2.406 participants (44 % male; mean age 28 ± 16; 67 % minority racial/ethnic group) were screened at 290 mobile drives” [29]. • A total of 14 % had none, 26 % one, 33 % two, and 27 % three or more risk factors. A total of 72 % of teenage participants had at least one risk factor. “A total of 18 % of participants who were taking medications for risks were poorly controlled. A total of 15 % had newly identified risks. • A total of 711 participants completed follow-up survey: 21 % sought medical care, 51 % were motivated to change their lifestyle, 81 % were pleased with screening” [29]. • “48 % were more likely to donate, and 62 % recommended donation to friends and family because of the screening” [29]. • To determine the value of screening in the donor setting, a long-term study must be undertaken. • CVD screening can improve donor health and can work as an incentive to donate (improve donation rates).	• Not implemented structurally.
10.	(Lenhard, Maser, Kolm, Healy, & Seshadri. 2013) [6]	Diabetes (n = 26.425)	USA	Retrospective analysis	• Plasma Glucose levels was collected, and Body Mass Index (by using weight and length) was calculated of 26.425 US donors to detect diabetes mellitus.	• Many individuals donate blood, and the addition of a voluntary screening glucose test may prove to be a unique and cost-effective method to assist in identifying the large number of individuals that have hyperglycaemia but are not aware of this. • “Low-, moderate-, and high-risk groups were formed based on RPG levels (<140, 140–200, and >200 mg/dL)”[6]. • “The three risk groups were similar, except for the body mass index”The Body Mass Index of the participants varied: o Group 1: 28.1 ± 5.4 kg/m2 o Group 2: 29.9 ± 5.5 kg/m2 o Group 3: 32.7 ± 5.6 kg/m2 (p < 0.0001) [6]. • “Cost analyses showed that the mean cost to screen, per donor, was less than 1 dollar” [6].	• The study was conducted 6 months. • The program was implemented for several years. Today it unfortunately is not any more [67].
11.	(Kessler, Grima, Valinsky, Ortiz, Hillyer, Nandi, Jones & Shaz, 2013) [30]	Cardiovascular Disease Risk factors (n = 26.201)	USA	Feasibility study	• From November 2010 through June 2011 26.201 individuals presented to participating blood drives, of these 11.195 individuals were interested in participating in the program, 10,382 participants were eligible to donate and participate (40 % of all presenting donors) and 9435 participants had complete data sets for analysis [30]. • “CVD risk screening was offered to donors at selected mobile drives in a large metropolitan area” [30]. • “Risk factors were determined by donor questionnaire, laboratory testing (total cholesterol, HDL levels and haemoglobin A1c), and blood pressure measurement” [30]. • “Results were reported to participants via mail and website. A 60-day follow up web-based survey was sent to participants via email to assess the impact of the program on donor's behavior” [30].	• 9435 donors between “17–75 years old participated with the following risk factors: 61.3 % BMIs >25, 28,8 % high total cholesterol, and 31,4 % lower than recommended HDL levels” [30]. • “25.3 % of donors that responded to the follow up survey went to see their health care provider based on screening results and 9 % of these received new or modified treatment” [30]. • Blood donors tend to be seen as “healthier than the general population, but many still have CVD risk factors, particularly obesity. CVD screening can be successfully used to make donors aware of this important health information and some donors act on this information” [30]. • Kessler et al. (2013) found out that 33.0 % of the blood donors in the pilot program had one, 27,3 % had two and 17,4 % had three or more risk factors.	• Pilot program
12.	(Gore, Eason, Ayers, Turer, Khera, de Lemos, McQuire, Sayers, 2014) [40]	Diabetes screening (n = 14.850)	USA	Cross-sectional study	• “HbA_1c_ was measured in 14.850 donors 16–19 years old during school blood drives conducted between September 1, 2011 and April 30, 2012” [40]. • The cohort included 48.7 % girls, 54.7 % individuals of European descent, 3.5 % individuals of African descent, 24.6 % individuals of Latin-American descent, 2.3 % individuals of Asian descent, and 15 % participants of other/unknown race/ethnicity [40].	• Among the donors in the study of Gore et al. (2014) there was a higher amount of prevalence of undiagnosed diabetes than the regular US population. • “Only 11 % of the donors retrieved their HbA1c results” [40]. • “The HbA1c level was above the diabetes threshold in 94 donors (0.6 %), and in the prediabetes range in 1.479 donors (10 %) [40]. • “There were no significant age differences across HbA1c categories (Ptrend 5 0.52), but a larger proportion of boys versus girls (16.5 vs. 5.0 %, P, 0.001) had HbA1c levels ≥5.7 % (including both the prediabetic and the diabetic range)” [40]. • “There were also marked differences in the prevalence of HbA1c levels ≥5.7 % across race/ethnic groups, with over-representation of individuals of African and Asian descent in the elevated HbA1c groups” [40]. • The HbA1c level was ≥5.7 % in: o 53.4 % of individuals of African descent, o 20.9 % of individuals of Asian descent, o 10.7 % of individuals of Latin-American descent, o 7.5 % of individuals of European descent (P, 0.001 for individuals of European descent vs. every other race/ethnic group)” [40]. o Boys: 16,5 % o Girls: 5,0 %.	• Not yet • Gore et al. (2014) mention that this is a pilot program. Despite it being a pilot program, the authors mention that blood banks could be a unique and efficient portal for prevention and health screening [40].
13.	(Gore, Eason, Ayers, Turer, Khera, de Lemos, mcGuire & Sayers, 2015) [39]	Diabetes screening (n = 31.546)	USA	Prospective, cross-sectional study	• This published study reported the results of the program's full implementation from a voluntary screening program including “31.546 consecutive volunteer blood donors, 16–19 years of age, who donated blood during school blood drives between September 1, 2011 and December 21, 2012 in Texas” [39].	• Of the donors included in the study, 11.0 % was in the prediabetes range [39]. • Gore et al. (2015) increased the scope of the pilot study in 2013 by expanding the numbers of participants. • This study confirms the findings of Gore et al., 2013, namely; abnormal HbA1C values are common in adolescent volunteer blood donors. • “The prevalence of elevated HbA1C was 11.5 %, including 11.0 % in the prediabetes range (HbA1C 5.7 %–6.4 %) and 0.5 % in the diabetes range (HbA1C ⩾ 6.5 %)”[39]. • The HbA1c level was ≥5.7 % in: o 32.7 % % of African descent, o 19.7 % of Asians descent, o 13,1 % of Latin American descent, o 8,0 % of European descent, o Boys: 15,7 %, o Girls: 7,9 % [39]. • “The prevalence of elevated HbA1C was higher in boys compared with girls and was especially high in racial/ethnic minorities” [39] compared to individuals of European descent [39].	• Yes
14.	(Samad, Yong, Mahendran, 2015) [31]	Diabetes screening (n = 211)	Malaysia	Short communication/cross-sectional study:	• The authors aimed to evaluate the feasibility of conducting routine diabetes screening in blood donation campaigns in Malaysia. • 211 blood donors were screened for diabetes [31]. • “The median age of donors was 45 years” [31].	• “The mean RCBS among blood donors was 6.0 (+1.9) mmol/L. 168 (79.6 %) donors were having normal RCBS, without diabetes mellitus” [31]. • “Further investigation on 43 (20.4 %) donors with RCBS ≥7.8 mmol/L indicated that 10 (5.0 %) donors had diabetes whereas 5 (2.5 %) donors had prediabetes” [31].	• Not mentioned in the study if its structurally implemented. • “Random blood sugar testing is easy, inexpensive and convenient” [31]. • “Neither an additional finger prick nor additional healthcare personnel is required for diabetes screening, as finger prick tests for haemoglobin level and ABO grouping of donors are performed routinely before blood donation” [31]. • Furthermore, diabetes screening requires only a mere additional 10 s to provide reliable information about a donor's diabetes status” [31].
15.	(Levi, 2015) [41]	Diabetes screening (n = 106)	Brazil	Letter to the editor	• From August 2012 up to April 2014, the blood bank of the Albert Einstein Hospital in Sao Paulo, Brazil, replaced blood donor screening for abnormal Hb levels using a traditional “agar-gel electrophoresis with an automated high-performance liquid chromatography (HPLC) method "[41]. Among 16,944 donors, the frequency of abnormal Hb levels was observed [41]. • The new method gave the opportunity to also measure HbA1c next to Hb levels. They retrieved HbA1c data from 6989 blood donations from 5790 donors during a period of 6 months (August 2012–February 2013).	• Levi (2015) found 106 donors with HbA1c values of 6.5 % or more, representing 1.8 % of the donations [41].	• Yes • They found HPLC advantageous since it is a fast and accurate automated procedure, it provides additional information such as HbA1c, and it allows the use of primary tubes, thus permitting electronic sample tracking.
16.	(Hao, McAvoy, Wickberg, Kerrigan, Kreiger, Sikavi, Swift, Frenette, Carney & Fung, 2016) [32]	Blood pressure (n = 835)	USA	Retrospective cohort study	• 1200 surveys were distributed at blood donation sites during the period of October 10, 2012 to October 26, 2012.	• Out of 839 survey responses received, 688 respondents reported their BP in the following categories, normotensive range: 46,9 %, pre-hypertensive range: 41,7 % and hypertensive range: 11,3 %” [32]. • “Notably, of donors with hypertensive range readings, 45 % reported no known history of hypertension” [32]. • “After reading the hypertension pamphlet almost 64 % of donors found it valuable, while almost 39 % did not” [32].	• Although an opportunity exists for increasing hypertension awareness during blood donation [32], the program is not yet implemented full scale in Vermont by the American Red Cross [68].
17.	(Anghebem-Oliveira, Costa, Baldanzi, Schmitt-Mansur, Picheth & Rego, 2017) [33]	Diabetes screening (n = 5.600)	Brazil	Cross-sectional study	• Out of 5600 candidates, 635 donors were included and participated in the pilot.	• “0,5 % of the donors had HBA1c levels suggestive of DM, and 57 donors (9 %) had levels associated with pre-DM. Regarding the risk of developing DM in 5 years, 111 donors were classified at moderate risk and 10 donors were classified at high risk” [33].	• Not yet • Blood banks could participate in DM screening, benefitting the public health care in Brazil [33].
18.	(Jackson, Keeton, Eason. Ahmad, Ayers, Gore, McGuire, Sayers, Khera, 2019) [34]	Cholesterol screening (n = 8.000.000)	USA	Cross-sectional analysis	• Between January 2002 and December 2016 about 8 million blood donors between the age of 16 years and older who donate blood to the Carter BloodCare located in Texas (US) were screened on total non-fasting cholesterol levels [34].	• Jackson et al. (2019) found that “in this study of 1.178.102 individual blood donors, 3473 individuals met the criteria for familial hypercholesterolemia” [34].	• Yes
19.	(Goel, Kessler, Nandi, Ortiz, Hillyer & Shaz, 2019) [35]	Cardiovascular Disease Risk (n = 570)	USA	Prospective controlled trial	• Study was a three-arm prospective controlled trial. “The primary objective was to determine if double versus routine incentive points led to improvement or maintenance of CVD risk profile assessed using self-reported changes "[35].	• “Positive donor reinforcement (double vs. routine points) resulted in better self-reported health maintenance behaviour and increased donation rates” [35]. • 570 total donors: group 1 (n = 290), group 2 (n = 134), group 3 (n = 146) [35]. • Positive behavioral changes were found in group 2 (62.0 %) en group 3 (71.4 %) [35]. • Group 2 increase in reading food labels (67.7 %–77.5 %) and group 3 (60.9 %–79.1 %) [35]. • Group 3 reported an exercise increase (Baseline: 52,9 %) and final 68.3 %) [35]. • The donations (median number of donations pro year): o Group 1: (4.4 [IQR, 2.7–8.0] vs. baseline 4.4 [IQR, 2.5–6.0] donations/year; p < 0.001). o Group 2: 2 (5.6 [IQR, 4.2–10.5] vs. baseline 4.9 [IQR, 3.5–10.2]). o Group 3: enrollment (6.8 [IQR, 4.3–12] vs. baseline 4.6 [IQR, 3.2–7.1] donations/year) [35].	• Yes
20.	(Agarwal, Gautam, Pursnani, Jain, Singh, Singh & Parihar, 2020) [42]	Diabetes (n = 1208)	India	Cross-sectional study	• Agarwal et al. (2020) studied the screening of 1.208 voluntary blood donors for diabetes and diabetic nephropathy to evaluate the feasibility of diagnoses by blood banks [42]. • The screening was conducted by blood screening and a urine check to detect prediabetes and undiagnosed Diabetes Mellitus (DM) and diabetic nephropathy [42].	• “This study demonstrated that 31 % (13 out of 48) diabetics had evidence of diabetic nephropathy” [42].	• Not yet. • Agarwal et al. (2010) recommends implementation of screening “voluntary blood donors for diabetes can be implemented to diagnose undetected burden of diabetic nephropathy in population” [42].

The included studies were published over the years from 1996 until 2020, and included eleven cross-sectional studies, four retrospective studies, two status reports, one observational study, one prospective controlled trial, one feasibility study**.** We then organized the studies by theme to gain insight in the available health check interventions implemented in blood banks used with the objective to contribute to donors’ health, according to stage 5 of the extended Marksey and O ‘Malley framework.

### 3.1 Health screenings

The literature studies addressed a range of health screenings and health promoting interventions not related to the safety of the blood donation process. These interventions addressed, cardiovascular risk assessment [29,30,[35], [36], [37]], diabetes screening [31,33,40,42], hypertension screening [26,32], cholesterol screening [34,38], cancer screening [25] and obesity screening [27]. The focus of 5 articles was on the assessment of cardiovascular risk assessment among donors, and 4 of these articles focused on diabetes screening. The studies highlighted the use of interventions assessing potential health risks and described how blood banks can contribute and initiate these preventive measures [26,28,[36], [37], [38]].

### 3.2 Community-based health intervention

Blood banks could be identified as portals for community-based health interventions, offering a highly efficient way of conducting large-scale public health promoting activities [5,28]. Traditionally, blood banks test all donors to ensure donor and blood safety. The literature showed that blood banks promoted public health and serve as a platform for offering additional health interventions [5,28], mainly using blood testing assessing cardiovascular health [12,13,34,38,39] and diabetes screening [31,33,40,42]. Blood banks can have a larger impact on community health by integrating health interventions in their daily activities, like obesity screening [27]. These initiatives, which involve the identification of risk factors within the donor population and the provision of essential health information, contributed to the overall well-being of individuals well beyond the donor community [5,29,38,40,43].

### 3.3 Health care context, tailoring and demographic

Studies showed that interventions of blood banks must be relevant to the country where they will be implemented. For example, diabetes is common in South Asia [31,42], thus blood banks can play a major role by early recognition of individual blood donors at risk of (pre-)diabetes to ensure deferral and (earlier) treatment [31,33,41,42].

Other factors that should be taken into consideration while designing interventions are age and gender differences. Some studies mention the importance of direct contact between physicians and donors in cancer screening among older male donors [25]. Other studies mention that offering optional screening tests may strengthen the relationship between donors and blood banks, thereby increasing and securing the blood supply, which benefits both donors and the community [25,29].

### 3.4 Practical implications for implementation

The literature showed that implementation of health screening interventions on a broader scale is limited [34,35,39,41]. One of the main barriers to implementation of health initiatives showed to be staff enthusiasm [28]. These initiatives might be seen as disruptive or not rightly fitting with regular care for donors [28]. It may also require new skills or training [5]. There is a need for blood banks to collaborate with stakeholders in the health care landscape to ensure the development of interventions to improve public health followed by the implementation for these interventions on a large scale [29].

### 3.5 Expert consultation

In line with the guidelines proposed by Levac et al. (2010) [20], next to scoping the literature, experts were consulted to obtain more insight in health initiatives implement by blood banks worldwide. In some blood banks in Germany, Japan [44], Lithuania, South Africa [38,45] and some parts of the USA [46] health check interventions are successfully implemented and obtained until now, with positive outcomes on donor health and public health, see Table 2. Other blood banks are still developing health initiatives, or did not implement the checks after successful pilots, or stopped after years of effective use, because of a lack of funding or showed not to meet the donors’ needs, or a combination of both. The specific needs of donors' may depend on the context of the bloodbank and should be assessed. Following donors’ needs also in how they prefer to receive their own health data after assessment, for example in a letter or online.

**Table 2:** Expert consultations (organized alphabetically by country).

Country	Blood bank	Health intervention(s)
Germany	German Red Cross-Blutspendedienst Nord-Ost	Since 2008 to this moment, two blood banks in German federal states of Hamburg, Berlin, Brandenburg, Saxony, and Schleswig-Holstein have been conducting additional health checks to donors. These health checks provide information about the blood pressure, haemoglobin, cholesterol, uric acid, and creatinine levels of whole blood donors [48].
Japan	Japanese Red Cross Society (JRCS).	Since 1982 JRCS have been administering a service to give information back to their donors, with the goal to improve their health by doing so [44]. First, they have been informing the donors with the results of their biochemical tests related to the donation process. And since 2009 JRCS has been screening donors for diabetes [44].
Lithuania	Blood Center of Vilnius University Hospital Santaros klinikos	At the Blood Center, blood donors can make a choice out of four packages of blood screening tests (lipid profile, liver enzymes and kidney function, vitamin D and Ferrin. By offering a choice the blood bank wants to attend the individual donor's needs [47].
Netherlands	Sanquin Blood Supply Foundation	Sanquin Dutch Blood Supply launched their new strategy ‘For Life' in 2022. Sanquin is currently exploring how they can promote donors’ health and create (more) reciprocity with their donors by market research among donors, and piloting.
USA	Carter BloodCare in North Texas	In the USA, HbA1c and total cholesterol were measured by Carter BloodCare in North Texas [34,39]. While screening for HbA1C was only offered as part of a now-completed research study (Dr. Maria Gore, personal communication, May 4, 2023), non-fasting total cholesterol continues to be measured in all Carter BloodCare donors [46].
USA	Blood Bank of Delmarva (BBD)-Christiana Center	BBD has successfully implemented a diabetes screening program and maintained for many years; many new cases of diabetes were detected (USA) [68]. They stopped screening.
USA	The New York Blood Bank Center	The New York Blood Bank Center stopped their cardiovascular screening and cholesterol screening for blood donors for a variety of reasons. At the time there was no electronic way to identify donors who wanted to be in the program on an ongoing basis. For the donors involved it seemed that not many looked to the blood donation experience for this type of health information. Ability to identify donors electronically who enrol in the program desiring ongoing screening would likely increase involvement, but not necessarily change the benefit of receiving this information or increase the rate of donation [70].
USA	American Red Cross in Vermont	Although “an opportunity exists for increasing hypertension awareness during blood donation” [32], this program is not yet implemented full scale in Vermont by the American Red Cross [69].

## 4 Discussion

In this review we showed health interventions are successfully implemented [44,46,47]. Blood banks as portals for community-based health: blood banks are efficient platforms for large-scale public health promotion activities, benefiting donors and the community [5,29,38,40,43]. We showed that structurally implemented health checks provide information on health indicators as lipide profile [46,48], diabetes screening [44], and vitamin D-levels [46]. The most common health interventions we included focused on cardiovascular risk assessment [29,30,[35], [36], [37]] and diabetes screening [5,28,31,33,40,42]. We identified that these interventions can be implemented with successful outcomes on donor health and public health, because healthy blood donors (do) have health risk factors [34,40,42], therefore they benefit from early screening [36,37] and create awareness and counselling by blood banks [5,27].

This study has limitations. The study did not include articles that did not focus on donors' health, like the use of donated personal (genetic) data to contribute to research [[49], [50], [51], [52]] and articles that focused on exploring the interaction between genetics and lifestyle factors of donors [53]. We also did not include articles that focused on the donors' willingness to donate because of receiving health checks. This may be a limitation because the health incentives in these articles may be potential benefits to donor health and the donated health data can improve donor health as well [54]. By excluding these articles, this study may not provide a comprehensive understanding of potential positive effects on using donors’ health data or how health initiatives might positively influence their health. A scoping review on the use of health data in general might be beneficial to get a comprehensive overview of all health promoting activities by blood banks. As noted by Brien et al. (2010) [55] the absence of quality assessment can make it difficult to interpret the results of scoping studies. In line with this, according to Grant and Booth (2009) [56], if the quality of a study is not assessed, it can be difficult to apply the knowledge in practice. We therefore evaluated the quality of the articles by using critical appraisal tools (see Appendix 2), even though our research questions did not directly focus on assessing article quality.

We provide several recommendations for policy and practice. Health interventions should suit the countries or regions’ needs. Prevalent conditions necessitate pro-active screening within donor populations. Plus -on regard to the legislative support and collaboration-there is a need for blood banks to collaborate with stakeholders in the health care landscape to ensure successful implementation on a large scale [28]. Some health screenings tests are used in practice by applicable laws, for example periodical preventive health screening of employees must be applied by all Dutch companies [57]. In the same way we would like to care for our blood donors. We believe therefore that collaborations between blood banks and local and national politics, health insurance companies, pharmaceutical companies, health foundations and general practitioners are needed to not only implement health initiatives on a large scale, but also sustain these initiatives. Besides that, one of the main barriers to implementation of health initiatives is staff enthusiasm [58]. These initiatives might be seen as disruptive [28]. It may also require new skills or training [5]. So, overcoming staff resistance is crucial for successful implementation. As are ethical concerns. Some ethical concerns exist (within staff) around remuneration for blood donors [59]. As monetary remuneration provides a higher risk for patients [60], there is no such evidence however that blood donors see health checks as monetary remuneration. In fact, health initiatives can work as an incentive [[61], [62], [63]]. Because the blood supply in many countries does not meet their needs [64], implementing health initiatives could be an option to reach a sufficient blood and plasma supply. In many countries worldwide remuneration for donation is by law forbidden. Also, due to a lack of non-monetary remuneration blood donation, blood supply in a number of countries still depends on (remunerated) plasma from the USA [65]. Countries that are by law bound to non-monetary remuneration blood donation buy USA plasma [65,66]. Thus, every country should build its own ethical standards [67].

We offer recommendations for future research. Research could be conducted in the context of the country where health checks are being implemented, as outcomes may vary per healthcare system. There is no established evidence that results of health checks outside the communicated normal ranges causes concern for the donors. Future research can explore the interventions directly at improving the use of extended health checks by tracking whether donors seek medical attention and how they navigate the healthcare system effectively, such as by attending primary healthcare physicians or other healthcare professionals. Moreover, there is no literature suggesting that health checks, perceived as remuneration, attract individuals who otherwise be deferred. Further research could explore this potential risk.

**Fig. 2:**
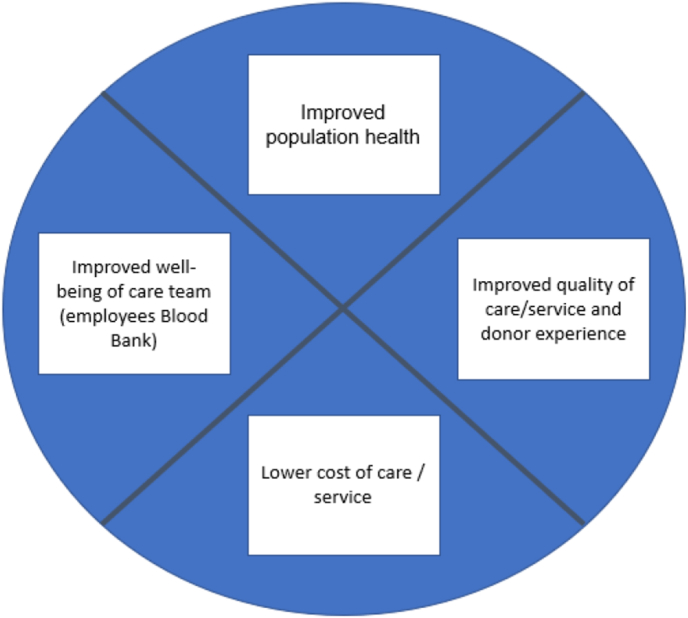
Quadruple aim (adapted, Bodenheimer and Sinsky, 2020).

In closing, implementing health initiatives should focus on employee experience, part of a quadruple aim approach, moreover to better quality of life of both the donor and society in general, better quality of service and cost-effectiveness (derived from Bodenheimer & Sinsky, 2014) [18], see Fig. 2.

## 5 Conclusion

This review highlights the implementation of health initiatives by blood banks, focusing on improving well-being and preventing diseases. These initiatives have the potential to act as gateways for community-based interventions and possibilities for enhancing both the blood supply and individual health. The effectiveness of these interventions is contingent upon the context of each blood bank and its target donor group. Therefore, tailoring interventions to align with specific contexts and facilitating factors is crucial for optimizing health promotion efforts tailored to the diverse needs of different donor groups.

## CRediT authorship contribution statement

**Linda J.H. Marx:** Writing – original draft, Methodology. **Suzanna M. Van Walraven:** Writing – original draft, Writing – review & editing. **Ruben van Zelm:** Writing – review & editing. **Barbara Sassen:** Writing – review & editing, Methodology.

## Ethics approval

As this is a review and data was obtained from open databases, no ethical approval was required.

## Data availability

You can contact the corresponding author for additional information.

## Declaration of competing interest

The authors declare that they have no known competing financial interests or personal relationships that could have appeared to influence the work reported in this paper.

## Figures and Tables

**Table:** 

How can blood banks contribute to the health of blood donors using prevention and health promotion activities?			Cinahl	Embase	PubMed	Web of Science
	Key words	Search Strings	26-May-2023	26-May-2023	26-May-2023	26-May-2023
Problem or donor	The patient or client in this search is "Blood Donors" or any term related to blood donors, such as "blood donor∗". The search aims to explore the role of blood banks in promoting public health and the health of blood donors.	Blood Donor OR Blood Banks OR Blood Center OR Blood Centre	8,707	94,804	28,810	4,485
Intervention	Intervention: The intervention being investigated is related to the concepts of "Role," "Involvement," "Contribution," "screening," "activit∗," and "intervention∗". The search is interested in understanding how blood banks are involved in these activities or interventions.	Role OR Involvement OR Contribution OR screening OR activit∗ OR intervention∗	1,706,056	11,123,952	7,825,006	1,305,022
Control	There is no control measure. The absence of any interventions or health initiatives is the control in this case.	None	Not applicable	Not applicable	Not applicable	Not applicable
Outcome	The outcome of interest is related to public health and the health of blood donors. The search includes terms like "Health Promotion," "Prevention and Control," "Public Health," "Health Education," "Primary Prevention," "Prevention," "Donor Health," "Donor Care," and "Donor Wellbeing". The goal is to explore the impact of blood banks on these outcomes	Health Promotion OR Prevention and Control OR Public Health OR Health Education OR Primary Prevention OR Prevention OR Donor Health OR Donor Care OR Donor Wellbeing	958,400	5,740,233	191	130,972
		Hits	506	52	191	508

**Table:** 

Publication	Appraisal tools	Checklist of items						Overall conclusion
		Abstract/Title	Methods	Results	Discussion	Conclusion	Other information	
Davey, 2006 [5]	Expert opinion	Good	Good	Good	Not described	Not described	Not described	Good
Shaz, Kessler, Hillyer, 2012 [28]	Expert opinion	Good	Good	Good	Not described	Not described	Not described	Good
Levi et al., 2015 [41]	Expert opinion	Good	Partially described	Good	Not described	Good	Good	Good
Hart et al., 1996. [25]	STROBE-cohort, case-control, and cross-sectional studies (combined)	Good	Mediocre	Good	Good	Good	Good	Good
Longo et al., 2007 [36]	STROBE-cohort, case-control, and cross-sectional studies (combined)	Good	Good	Good	Good	Good	Good	Good
Dell’Anna et al., 2010 [37]	STROBE-cohort, case-control, and cross-sectional studies (combined)	Mediocre	Good	Mediocre	Good	Good	Good	Good
Eason et al., 2011 [38]	STROBE-cohort, case-control, and cross-sectional studies (combined)	Good	Good	Good	Good	Good	Not described	Good
Kessler et al., 2012 [29]	STROBE-cohort, case-control, and cross-sectional studies (combined)	Good	Good	Good	Good	Good	Good	Good
Murphy et al., 2012 [27]	STROBE-cohort, case-control, and cross-sectional studies (combined)	Good	Good	Good	Good	Good	Good	Good
Mathai, Vasu, Subhadramma, 2012 [26]	STROBE-cohort, case-control, and cross-sectional studies (combined)	Good	Good	Good	Good	Good	Good	Good
Lenhard et al., 2013 [6]	STROBE-cohort, case-control, and cross-sectional studies (combined)	Good	Good	Good	Good	Good	Good	Good
Kessler et al., 2013 [70]	STROBE-cohort, case-control, and cross-sectional studies (combined)	Good	Good	Good	Good	Good	Good	Good
Gore et al., 2014 [40]	STROBE-cohort, case-control, and cross-sectional studies (combined)	Good	Good	Good	Good	Good	Good	Good
Gore et al., 2015 [39]	STROBE-cohort, case-control, and cross-sectional studies (combined)	Good	Good	Good	Good	Good	Good	Good
Samad, Yong, Mehandran, 2015 [31]	STROBE-cohort, case-control, and cross-sectional studies (combined)	Good	Good	Good	Good	Good	Good	Good
Hao et al., 2016 [32]	STROBE-cohort, case-control, and cross-sectional studies (combined)	Good	Good	Good	Good	Good	Good	Good
Anghebem-Oliveira et al., 2017 [33]	STROBE-cohort, case-control, and cross-sectional studies (combined)	Good	Good	Good	Good	Good	Good	Good
Goel et al., 2019 [35]	STROBE-cohort, case-control, and cross-sectional studies (combined)	Good	Good	Good	Good	Good	Good	Good
Jackson et al., 2019 [34]	STROBE-cohort, case-control, and cross-sectional studies (combined)	Good	Good	Good	Good	Good	Good	Good
Agarwal et al., 2020 [42]	STROBE-cohort, case-control, and cross-sectional studies (combined)	Good	Good	Good	Good	Good	Good	Good

## Appendix 1

Boolean Phrases:

CINAHL:

((("Blood Donors" OR "blood donor∗") OR ("Blood Banks" OR "blood bank∗" OR "blood center∗" OR "blood centre∗"))

AND

("Role" OR "Involvement" OR "Contribution" OR "screening" OR "activit∗" OR "intervention∗")

AND

(("Health Promotion" OR "health promotion") OR ("Prevention and Control" OR "prevention and control") OR ("Public Health" OR "public health") OR ("Health Education" OR "health education") OR ("Primary Prevention" OR "primary prevention") OR ("Prevention" OR "prevention") OR ("Donor Health" OR "Donor Care" OR "Donor Wellbeing"))

Embase (modified with EMTREE terms):

(('Blood donors'/exp OR 'blood donor∗') OR ('Blood banks'/exp OR 'blood bank∗' OR 'blood center∗' OR 'blood centre∗')

AND

("Role" OR "Involvement" OR "Contribution" OR "screening" OR "activit∗" OR "intervention∗")

AND

('Health promotion'/exp OR 'prevention and control'/exp OR 'public health'/exp OR 'health education'/exp OR 'primary prevention'/exp OR 'prevention'/exp) AND ('Donor health' OR 'Donor care' OR 'Donor wellbeing'))

Pubmed:

((“Blood Donors”[Mesh] OR “blood donor∗[tiab] OR ‘Blood Banks”[Mesh] OR “blood bank∗” [tiab] OR “blood center∗” [tiab] OR “blood centre∗” [tiab])

AND

("Role"[TIAB] OR "Involvement"[TIAB] OR "Contribution"[TIAB] OR "screening"[TIAB] OR "activit∗"[TIAB] OR "intervention"[TIAB])

AND

("Health promotion" [Mesh] OR "health promotion" [tiab]) OR ("Prevention and control" [Mesh] OR "prevention and control" [tiab]) OR ("Public health" [Mesh] OR "public health" [tiab]) OR ("Health education" [Mesh] OR "health education" [tiab]) OR ("Primary prevention" [Mesh] OR "primary prevention" [tiab]) OR ("Prevention" [Mesh] OR "prevention" [tiab]) ("Donor health"[tiab] OR "Donor care"[tiab] OR "Donor wellbeing"[tiab]))

Web of science

TI=(("Blood Donors" OR "blood donor∗") OR ("Blood Banks" OR "blood bank∗" OR "blood center∗" OR "blood centre∗"))

AND

TI= ("Role" OR "Involvement" OR "Contribution" OR "screening" OR "activit∗" OR "intervention∗")

AND

TI=(("Health Promotion" OR "health promotion") OR ("Prevention and Control" OR "prevention and control") OR ("Public Health" OR "public health") OR ("Health Education" OR "health education") OR ("Primary Prevention" OR "primary prevention") OR ("Prevention" OR "prevention") OR ("Donor Health" OR "Donor Care" OR "Donor Wellbeing"))

Or

TS=("Blood Donors" OR "blood donor∗" OR "Blood Banks" OR "blood bank∗" OR "blood center∗" OR "blood centre∗") AND TS=("Role" OR "Involvement" OR "Contribution" OR "screening" OR "activit∗" OR "intervention∗") AND TS=("Health Promotion" OR "health promotion" OR "Prevention and Control" OR "prevention and control" OR "Public Health" OR "public health" OR "Health Education" OR "health education" OR "Primary Prevention" OR "primary prevention" OR "Prevention" OR "prevention" OR "Donor Health" OR "Donor Care" OR "Donor Wellbeing")
